# The Role of Neuroplasticity in Improving the Decision-Making Quality of Individuals With Agenesis of the Corpus Callosum: A Systematic Review

**DOI:** 10.7759/cureus.26082

**Published:** 2022-06-19

**Authors:** Leopoldo Mandic Ferreira Furtado, Henriqueta Morais Bernardes, Felipe Alexandre de Souza Félix Nunes, Carlos Alberto Gonçalves, José Aloysio Da Costa Val Filho, Aline Silva de Miranda

**Affiliations:** 1 Pediatric Neurosurgery, Vila da Serra Hospital, Nova Lima, BRA; 2 Department of Technological Innovation, Federal University of Minas Gerais, Belo Horizonte, BRA; 3 Center for Research and Graduate Studies in Business Administration, Federal University of Minas Gerais, Belo Horizonte, BRA; 4 Neuroscience and Management Science, Federal University of Minas Gerais, Belo Horizonte, BRA; 5 Neurosurgery, Vila da Serra Hospital, Nova Lima, BRA; 6 Department of Morphology, Federal University of Minas Gerais, Belo Horizonte, BRA

**Keywords:** functional connectivity, commissures, decision-making, neuroplasticity, corpus callosum agenesis

## Abstract

Although individuals with agenesis of corpus callosum (ACC) possess intelligence coefficients within regular parameters, current studies have demonstrated decision-making compromise and potential negative social consequences. Furthermore, alternative pathways regarding brain connectivity in acallosal patients combined with cognitive therapy that would potentially mitigate such difficulties. Therefore, this study aimed to examine the current state of the art regarding brain foundations in the role of neuroplasticity by improving the decision-making quality in ACC.

A systematic revision of literature was performed including studies conducted on non-syndromic ACC individuals and analyzing the impact of the potential role of neuroplasticity on the decision-making published to date. Studies with patients who underwent callosotomy were excluded. Experimental studies performed on animal models were included.

During this period, 849 studies were identified; among them, 11 were eligible for qualitative analysis. Despite the paucity of evidence on this matter, patients with ACC present considerable decision-making difficulties mainly due to the functional connectivity impairment in the frontal lobes. Moreover, neuroplasticity was characterized by increased anterior commissure width as compared with controls. Notwithstanding, no studies were conducted on cognitive therapists managing this type of disease.

Although the reorganization of inter-hemispheric bundles on anterior commissure has demonstrated the main natural neuroanatomic strategy in ACC, further evidence will be needed to clarify whether cognitive stimulus could improve the decision-making quality.

## Introduction and background

Congenital abnormalities of the corpus callosum (CC) are brain malformations including a partial or complete absence of CC and hypoplasia, with incidence ranging from 1.47 per 10,000 in the general population according to the Emilia Romagna Italy report, i.e., 1 of 4,000 live births in agenesis of the corpus callosum (ACC) in other studies [1-4]. Although a set of genetic malformations were associated with ACC such as Chiari II malformation, Anderman syndrome, Muscle-eye brain disease, Aicardi syndrome, and other diseases, the cause of ACC is not determined in 75% of patients, which are considered isolated or primary ACC [4, 5]. Furthermore, several neurocognitive and emotional impairments have been consistently observed in patients with primary ACC, such as abstract reasoning, problem-solving, generalization, category fluency, alexithymia, and recently reported decision-making ability [4, 6].

The decision-making behavior has been considered one of the main pillars of neurocognition and is important during daily choices or taking resolutions demanding more sophisticated skills, such as planning and reasoning [7]. Neuroanatomically, this process depends on specific brain regions, such as the anterior segment of the cingulum, medial prefrontal cortex, striatum, and CC [8, 9]. Based on the main and larger brain commissure, the CC is composed of approximately 190 million axons and participates not only in the cognitive process but also in the integration of information between the hemispheres. Its function allows the activation of homologous regions or its inhibition in some instances to warrant the effectiveness of brain function [4, 10, 11]. In Schoenemann et al. [12] proposed comparative study between humans and other primates, the brain’s complexity increases from its connectivity and the decision-making understandable as a higher-order expression by combining the following abilities: attention, memory, and judgment depending on the ultimate brain network.

The brain’s functional connectivity associated with the CC could be compromised by iatrogenic or congenital situations, such as callosotomy and ACC, respectively, and these have been converted into valuable models to understand the physiology of this commissure and the potential effects of rehabilitative interventions. Interestingly, as serious neurological consequences occurred if complete callosotomy is performed, which would evoke the so-called split-brain condition, lesser neurological compromise is normally observed on the ACC.

Although the importance of the CC on the brain’s connectivity was proven, some controversies remained because individuals who underwent partial callosotomy displayed no differences in inter-hemispheric neuronal synchronicity [13], and an acceptable explanation is the replacement’s effect on other commissures, such as the anterior, posterior, habenular, and hippocampal regions [4]. On the other hand, patients with ACC presented decision-making impairments and difficulties in solving novel problems [14] perhaps due to the remaining compromise of the associated brain regions in congenital aberrations of CC although the compensation was determined based on the anterior commissure’s width [9, 15].

Given that the decision-making of patients with ACC was compromised, a valid correlation should be established between neuroplasticity’s improvement and neurocognition in these patients. To our knowledge, this could be converted as a valuable guide for pediatric neurosurgeons and neurologists by talking to their fathers on how to proceed in a long-term plan in children diagnosed with ACC and the potential impact of early cognitive stimulation in improving high-level functions of the brain. Therefore, this study aimed to examine the current state-of-the-art effects of neuroplasticity on patients with ACC focusing on decision-making, and shedding light on further studies.

## Review

Methods

A systematic literature revision was performed by searching the data bank available on the PubMed and Web of Science and retrieving studies focusing on the ACC and its relationship with neuroplasticity and decision-making. The research process followed the Prisma protocol [16].

Inclusion Criteria

This systematic review considered all studies focusing on the pediatric population affecting the ACC in which the functional connectivity was examined using imaging strategies, such as functional magnetic resonance imaging (fMRI) and/or MRI with diffusion tensor imaging, neuroplasticity, and decision-making of individuals with ACC.

Moreover, were included the experimental studies in which ACC animal models were adopted and the brain structure using imaging or microscopic techniques was examined.

Clinical and experimental studies conducted to date were considered in this study. Furthermore, studies deemed for analysis were those published exclusively in the English language.

Behavioral tests, intelligence scales, and other types of neurocognitive assessments were performed. However, they were not considered inclusion criteria.

Exclusion Criteria

Human studies conducted neurocognitive tests alone without brain imaging or examining the brain structure were excluded from this review. Furthermore, genetic studies on animal models that examined the relationship between genes and ACC were also excluded. In addition, studies including patients who underwent surgical disconnection of the CC, case reviews, and syndromic cases were excluded. Therein, studies conducted on patients with primary ACC preferred for analysis over dysgenesis and hypoplasia of CC despite such malformations were considered.

Intervention or Exposure and Outcomes

Rehabilitative interventions if available in the literature were included in the analysis to improve decision-making in patients with primary ACC. The primary outcome was the functional impact of neuroplasticity on ACC, and secondary outcomes were neuroanatomic adaptations.

Search Strategy and Data Extraction

An electronic literature search was performed by two independent researchers who compiled the papers. The PubMed and Web of Science databases were used in this study using the following constructs: 'agenesis of corpus callosum' and 'decision-making'; 'agenesis of corpus callosum' and 'neuroplasticity'; 'corpus callosum' and 'decision-making'; and 'corpus callosum' and 'neuroplasticity'.

Results

After applying the aforementioned criteria, 849 studies were identified. Moreover, the following results were achieved from the constructs on PubMed: 'corpus callosum' and 'decision-making' (165 studies); 'corpus callosum' and 'neuroplasticity' (350 studies); 'agenesis of corpus callosum' and 'decision-making' (24 studies); and 'agenesis of corpus callosum' and 'neuroplasticity' (30 studies). On the Web of Science, the following results were observed: 'corpus callosum' and 'decision-making' (157 studies); 'corpus callosum' and 'neuroplasticity' (101 studies); 'agenesis of corpus callosum' and 'decision-making' (13 studies); and 'agenesis of corpus callosum' and 'neuroplasticity' (9 studies).

**Figure 1:**
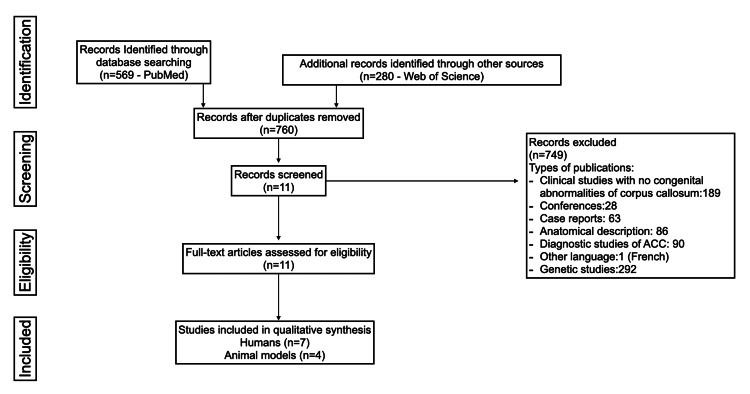
Flowchart of search mechanism based on the PRISMA-P 2015

Duplications and exclusions were performed, and finally, 11 studies were considered for qualitative analysis (Figure 1).

Discussion

Foundations of Neuroplasticity

Individuals who presented with stroke, brain trauma injuries, or were born with connectivity impairments have the potential to develop multifactorial adaptative changes allowing them to replace the compromised brain function [17-21]. The awareness of this phenomenon is not new and was firstly defined as “plasticity” by Willian James in 1890 and modified as “neural plasticity” by Jerzy Konorki in 1948 [22]. Currently, a wide understanding of neuroplasticity evolves on the notion of brain changes and adaptation as a product of interaction with the environment in a dynamic process that results in many recovery levels in the motor, behavioral, learning, and memory functions [18, 21, 22]. Although studies have initially indicated the association between neuroplasticity and gray matter, most importance was given to the white matter, such as the CC and internal capsule [23]. Conversely, molecular foundations of neuroplasticity include the synaptic dynamicity to suffer changes and in the role of molecules such as N-methyl-D-aspartate, nerve growth factor, brain-derived growth factor, and mitochondrial action [22]. Furthermore, neuroplasticity of the developing brain could be observed in behavioral functions and in ACC, with structures that will promote such an adaptation are the anterior commissure, posterior commissure, and the CC integrity on partial ACC [24]. In humans, the anterior commissure, with an average of 3.5 million axons on its structure, connected both the basal ganglia posterior superiorly to the septal nuclei and could increase by 10% of its width in patients with ACC [24, 25]. Therefore, the hypothesis of the natural absence of inter-hemispheric connectivity as observed in ACC could be replaced by another commissure is corroborated by a study in which normal connectivity was identified in the brains of patients with ACC employing the resting-state functional magnetic resonance [26].

Decision-Making and ACC

One of the major neurocognitive decision-making aspects is the ability to predict the future reward and choose a low gain in a short term, which is recognized as a delayed reward discount [8]. Individuals with the inability of such a perception have a trend to depict precipitated behaviors during the decision-making tasks and studies focusing on determining neural foundations to justify this process, mainly using the functional magnetic resonance task based on the correlation by activating several cortical and subcortical regions [27]. On one hand, some centers are evolved with cognitive control such as the dorsolateral frontal cortex and anterior segment of the gyri in the cingulum, whereas the reward assessment is related to the ventral striatum, orbitofrontal cortex, insula, and tegmental ventral area. Moreover, self-reflexive thought and targeted for future events are associated with the activation of the medial prefrontal cortex, the posterior segment of the cingulum, the temporoparietal junction, and medial and lateral regions of the temporal lobe [8]. Impulsive individuals and drug addiction or bipolar behavior showed lesser activity of the CC, and children with attention-deficit hyperactivity disorder have shown a reduced size of the CC rostrum and body [28-30]. Similarly, Magara et al. [31] observed impulsivity and hyperactivity behavior in mouse models with ACC.

Undoubtedly, a study on pathological conditions aids in the awareness of several complex brain functions, and the decision-making quality is better appreciated in selecting patients with punctual deficiencies in specific brain regions such as ACC. Furthermore, the assessment of these individuals shows alternative pathways of neuroplasticity. Overall, they have regular intelligence scores besides the decision-making difficulty, suggesting the major role of interconnectivity instead of the gray matter during this process. Interestingly, animal studies have contributed to the best understanding of CC function and in some instances, different species separated for millions of years demonstrated a curious decision-making process. Therefore, CC is a mammalian structure that increases as an evolutionary jump [32], and, therefore, birds have no CC and just a small anterior commissure, depicting simultaneous activity on both hemispheres during the decision-making process [33].

Owen et al. [34] analyzed the connectomic of patients with partial and complete absence of CC as compared with normal individuals and found alterations in the bilateral connectivity on the frontal-parietal regions, precuneus, posteromedial parietal region, and insula. Notwithstanding, they reported symmetrical preservations even with alterations on the special distribution as compared with the control group, deducting that patients with ACC have impaired associative cortex network.

Although patients with ACC have been demonstrated to have acceptable cognitive performance and can execute social tasks that demand reasoning or manual capacity, alterations associated with the anterior region of the insula could affect social behaviors requiring leadership. Such a statement is corroborated by Edelson et al. [35] who used functional MRI tasks based on the decision-making assessment associated with the leadership behavior and described lesser activation on the anterior insular region in individuals with more responsibility aversion, a quality considered to be associated with leadership.

One of the limitations of the studies was the method used to assess the connectivity in patients with ACC using resting-state functional MRI, in which the brain activity is not evaluated during the high demand and, therefore, just reveals the baseline brain activity based on fluctuations of blood oxygen consumption. Imaging studies were applied during tasks.

Brown et al. [1] studied the decision-making quality of patients with ACC and performed an experiment recruiting 40 individuals affecting the malformation presenting 26 years of mean age as compared with the control group with a mean age of 26 years. They observed the mean intellectual performance of 96.8 in patients with ACC based on the test “full-scale intelligence quotient” against 99.65 in the control group. To assess the decision-making ability, the “Iowa Gambling test” was used consisting of a game that used the punishment and monetary reward, evaluating abilities associated with the decision-making involved in the emotion and intuition; furthermore, novel situations demanding resolution were also presented. They reported a significant difference among the groups concerning the consistency of choices and the ACC group scored significantly lower than that of the control (p < 0.05), showing more tendency to make choices less consistent than that of normal individuals. More propensity was associated with more losses due to strategic mistakes that were not perceived in some situations, indicating that the big gain would mean big losses.

Perspectives of High-Order Brain Improvement

The difficulty of solving new problems and making the right decisions while balancing the pros and cons is likely related with the deal with excess information of the environment correctly. One plausible explanation is anatomic and associated with a smaller diameter of the anterior commissure, with the main path to neuroplasticity for this case, allowing the performance and processing of all information between the frontal hemispheres. Moreover, the lack of flexible thought attributed to patients with ACC and autism is related to the difficulty in processing different information for different decision-making, becoming more likely to choose based on the rigid mental process. Therefore, two possible strategies are required for this hypothesis: offering problems in a slow form and cognitive training to ameliorate the brain capacity for integration. To date, leadership specifically in the field of decision-making is still not evident. However, indirect evidence reinforced this impression of optimization of inter-hemispheric connectivity in the brain. For instance, the excess of social media use could worsen the integration between hemispheres mainly in the forceps minor of CC, whereas the stress experience with the application of tough tasks has proven to improve this integration [36, 37]. Nowadays, such a finding could be converted into lesser rehabilitative effectiveness considering the dissemination of smartphones and social media contemporarily.

One goal of the rehabilitative approach was proposed by the international classification of Functioning, Disability, and Heath considering the bio-psychosocial aspect of these patients, stimulating general functions and improving social integration, not only a specific function associated with CC has given more importance to the environment as a facilitator. Moreover, monitoring neuropsychological disturbances during school age is mandatory to explore the best neuroplasticity [5] (Tables 1 and 2).

**Table 1:** Research on humans with congenital alterations of the corpus callosum

Author/ Year	Sample size	Design	Results
Pelletier et al., 2011 [38]	ACC (n = 6) Controls (n = 6)	Retrospective	No difference of language lateralization in ACC compared to control groups. ACC shows a more bilateral pattern of activation than high-IQ participants.
Brown et al., 2012 [1]	ACC (n = 40) Controls (n = 26)	Retrospective	ACC had a lower overall net gain and fewer advantageous choices than controls.
Siffredi et al., 2019 [39]	21 children with ACC (13 complete, 8 partial) 30 controls aged 8–17 years.	Prospective	Anterior commissure was significantly larger in volume in children with ACC than controls (p = 0.027). Partial ACC and larger posterior commissure volume were associated with better orienting attention (p = 0 .035).
Siffredi et al., 2020 [40]	20 ACC 29 controls	Prospective	Children with ACC and controls showed a similar pattern of intra- and inter-hemispheric connectivity.
Szczupak et al., 2021 [41]	11 CC dysgenesis (five with ACC and six with hypoplasia)	Prospective	ACC has a scant number of inter-hemispheric connections but manages to maintain the full integrity of functional connectivity between the same cortical regions as healthy patients.
Shi et al., 2021 [42]	19 children ACC (13 with complete, 6 with partial) 29 controls	Prospective	ACC has structural strengthening of intra-hemispheric pathways as a neuroplastic response Regional variability in structural connectivity in children with ACC compared to controls.
Siffredi et al., 2021 [43]	20 children with CC dysgenesis 29 controls.	Prospective	Atypical bundles were observed in 30% of patients with ACC crossing via the anterior commissure 30% crossing via the posterior commissure vs. 6.9% of the controls

**Table 2:** Agenesis of the corpus callosum studies in animals included

Author/ Year	Sample	Design	Results
Magara et. al., 2000 [31]	Mice	Experimental study	Behavior alterations associated with acallosal mice
Vitral et al., 2006 [44]	Mice	Experimental study	Recovery of behavioral functions after prenatal damage associated with specific factors of local cortical circuitry organization. Changes on the inter-hemispheric cortical integration through the corpus callosum could promote relatively fixed cognitive dysfunctions, as those observed on performances that require strategies for decision-making
Edwards et al., 2020 [45]	Mice	Experimental study	MRI-based tractography approach combined with histological validation demonstrates that the structural rewiring cannot be explained purely by the absence of callosal connections.
Wittek et al., 2021 [33]	Pigeons	Experimental study	The simultaneous brain activity of birds during the decision-making

Limitations

Although this systematic review has compiled a wide range of studies regarding congenital aberrations affecting the CC in experimental and clinical studies to obtain an in-depth understanding of how neuroplasticity works in these patients, some limitations were observed. Only a few studies have focused on neuroplasticity in these patients, and only one study showed compromised decision-making in a well-delineated study. Furthermore, studies including primary acallosal patients are quite rare, making it impossible to draw a reliable meta-analysis. Nonetheless, this study shed light on important nuances regarding neuroplasticity, which should be considered by patients and physicians to stimulate the affected children early.

## Conclusions

Parents of children diagnosed with ACC should be informed of the expected decision-making difficulty and potential negative consequences during adulthood, despite normal levels of intelligence. Furthermore, an immediate recommendation from the cognitive therapist remains a theoretical potential benefit for the improvement of neuroplasticity.

Although the augmented size of anterior commissure constitutes the major neuroplasticity anatomical event in acallosal patients, no current evidence is available to recommend an efficient cognitive therapy to improve the decision-making quality by explaining the heterogeneity among patients. Further experimental studies will be needed especially in employing molecular markers to clarify this field of knowledge.
